# Arterial/venous thrombosis, fetal loss and stillbirth in pregnant women with systemic lupus erythematosus versus primary and secondary antiphospholipid syndrome: a systematic review and meta-analysis

**DOI:** 10.1186/s12884-018-1850-x

**Published:** 2018-06-07

**Authors:** Pravesh Kumar Bundhun, Mohammad Zafooruddin Sani Soogund, Feng Huang

**Affiliations:** 1 Department of Internal Medicine, the First Affiliated Hospital of Guangxi Medical University, Nanning, Guangxi 530021 People’s Republic of China; 2 Guangxi Medical University, Nanning, Guangxi 530027 People’s Republic of China; 3 Institute of Cardiovascular Diseases and Guangxi Key Laboratory Base of Precision Medicine in Cardio-cerebrovascular Diseases Control and Prevention, the First Affiliated Hospital of Guangxi Medical University, Nanning, Guangxi 530021 People’s Republic of China

**Keywords:** Systemic lupus erythematosus, Primary antiphospholipid syndrome, Secondary antiphospholipid syndrome, Pregnancy, Venous thrombosis, Arterial thrombosis, Fetal loss, Stillbirth

## Abstract

**Background:**

We aimed to systematically compare arterial/venous thrombosis, fetal loss and stillbirth in pregnant women with systemic lupus erythematosus (SLE), primary anti-phospholipid syndrome (PAPS) and secondary anti-phospholipid syndrome (SAPS).

**Methods:**

Online databases were carefully searched for relevant publications comparing SLE with PAPS and/or SAPS in pregnancy. Studies were included if: they compared SLE with APS [SLE versus PAPS or SLE versus SAPS or SLE versus PAPS and SAPS respectively] in pregnant women; and they reported specific adverse outcomes as their clinical endpoints including arterial/venous thrombosis, fetal loss and stillbirth. Risk ratios (RR) with 95% confidence intervals (CIs) were used as statistical parameters and the analysis was carried out by the RevMan 5.3 software.

**Results:**

A total number of 941 pregnant women were included: 556 were candidates of SLE; 200 were candidates of PAPS; and 185 were candidates of SAPS. APS was associated with a significantly higher risk of fetal loss (RR: 4.49, 95% CI: 2.09–9.64; *P* = 0.0001). In addition, stillbirth and arterial/venous thrombosis were also significantly increased with APS (RR: 6.65, 95% CI: 2.14–20.60; *P* = 0.001) and (RR: 3.95, 95% CI: 1.28–12.16; *P* = 0.02) respectively.

When patients with PAPS were compared with patients who suffered from SLE alone, fetal loss and arterial/venous thrombosis were still significantly higher with the former.

When SAPS were compared with SLE (without anti-phospholipid antibodies), arterial/venous thrombosis, stillbirth and fetal loss were still significantly higher with SAPS. However, no significant difference was observed in arterial/venous thrombosis and fetal loss between PAPS and SAPS.

**Conclusions:**

PAPS and SAPS were associated with significantly higher arterial/venous thrombosis, fetal loss and stillbirth in comparison to SLE. However, no significant difference was observed when PAPS was compared to SAPS.

## Background

Systemic lupus erythematosus (SLE) is an autoimmune disorder which affects a small population of women of childbearing age [1]. Even if research focusing on pregnant women with SLE was seldom carried out due to a limited number of similar patients who agreed to participate in research cohorts, requiring several decades to obtain a minimum number of participants, scientific medical reports which were successfully published have shown this disorder to significantly be responsible for adverse maternal and fetal outcomes [2]. However, newly published research has shown those adverse outcomes to further exacerbate by factors such as renal involvement, lupus nephritis, and anticardiolipin antibodies [3].

SLE has a complicated pathogenesis [4–6]. New scientific research has shown a clear association of SLE with anti-phospholipid syndrome (APS) [7], mainly secondary anti-phospholipid syndrome (SAPS). The co-existence of APS with SLE has often aggravated the course of the latter.

Primary anti-phospholipid syndrome (PAPS) is another rarely encountered autoimmune disorder affecting such patients.

Because studies comparing SLE with PAPS and SAPS are limited, research which are based on pregnant women with co-existing SLE and APS has seldom been possible.

SLE and APS (PAPS and SAPS) are associated with arterial and venous thrombosis as well as recurrent fetal loss. Nevertheless, it is not well known which one among these disorders is associated with the most severe consequences during pregnancy.

Since we have been able to extract some data from online databases, we aimed to compare arterial/venous thrombosis, fetal loss and stillbirth in pregnant women with SLE, PAPS and SAPS.

## Methods

### Searched databases

EMBASE (www.sciencedirect.com), MEDLINE database of medical research articles, and Google Scholar were carefully searched for relevant publications comparing SLE with PAPS and/or SAPS in pregnant women.

In addition, official websites of major rheumatology and maternity journals were also reviewed for relevant articles.

### Searched strategies

The following words/terms/phrases were used during the search strategy:

- Systemic lupus erythematosus, antiphospholipid syndrome and pregnancy;
- Systemic lupus erythematosus, antiphospholipid syndrome and maternal outcomes;
- Systemic lupus erythematosus, antiphospholipid syndrome and fetal outcomes;
- Systemic lupus erythematosus, primary antiphospholipid syndrome and pregnancy;
- Systemic lupus erythematosus, secondary antiphospholipid syndrome and pregnancy;
- Hughes syndrome and pregnancy;
- Autoimmune disorders and pregnancy outcomes;
- SLE, APS and pregnancy;
- SLE, APS and maternal outcomes;
- SLE, APS and fetal outcomes.

This search which was carried out in accordance to the PRISMA guideline [8], was restricted to English publications.

### Inclusion criteria

Studies were included if:

- They were randomized trials or observational studies comparing SLE with APS [SLE versus PAPS or SLE versus SAPS or SLE versus PAPS and SAPS respectively] in pregnant women;
- They reported adverse outcomes as their clinical endpoints; focusing mainly on arterial/venous thrombosis, fetal loss and stillbirth (major outcomes).

### Exclusion criteria

Studies were excluded if:

- They were review articles;
- They were case studies;
- They did not compare SLE with APS in pregnant women;
- They did not report adverse outcomes (at least arterial/venous thrombosis, fetal loss or stillbirth) as their clinical endpoints;
- They were replicated/repeated studies.

### Types of participants and main definitions

In this analysis, pregnant women with SLE alone, pregnant women with PAPS and pregnant women with SAPS (most of the time it was associated with SLE) were included.

SLE is defined as an autoimmune disorder which affects mainly women of child-bearing age. There is no exact cause of SLE, however, genetic and environmental factors have shown to be among the causes. Painful swollen joints, malar rash, oral ulcers, photosensitivity, renal and cardiovascular symptoms and inflammation are among its manifestations.

APS also known as Hughes syndrome is defined as an autoimmune disorder with the presence of anti-phospholipid antibodies and anticardiolipin antibodies, manifesting as arterial and venous thrombosis and pregnancy related complications as the common symptoms. PAPS implies that the disorder is not due to and not co-existing with other disorders. However, SAPS implies that the disorder has been caused secondary to another disease.

### Outcomes and definitions

Outcomes which were assessed through this analysis were:

- Arterial/venous thrombosis;
- Fetal loss which was defined as death of a fetus beyond ten weeks of gestation;
- Stillbirth which was defined as death of the fetus prior to delivery/at least after 28 weeks of gestation;
- Infants who were considered low for gestational age, that is, birth weight below the tenth percentile for the corresponding gestation;
- Premature or preterm delivery: defined as the termination of pregnancy with a live birth before 37th week of gestation.

Major outcomes were arterial/venous thrombosis, fetal loss and stillbirth, whereas the other outcomes were minor endpoints. The reported outcomes in patients with SLE versus PAPS and in patients with SLE versus SAPS were listed in Tables 1 and 2 respectively.

**Table 1:** Outcomes which were reported in participants with SLE versus PAPS

Studies	Outcomes reported	Types of participants
Paramo 2002 [11]	Fetal loss	SLE versus PAPS in pregnancy
Huong 2006 [12]	Fetal death, pre-eclampsia, arterial occlusion, arterial and venous thrombosis, premature	SLE versus PAPS in pregnancy
Muñoz Rodriguez 2000 [13]	Miscarriage, arterial and venous thrombosis, thrombocytopenia	SLE versus PAPS in pregnancy
Tarr 2007 [14]	Fetal loss, and thrombosis	SLE versus PAPS with SLE in pregnancy
Cervera 2013 [15]	Pre-eclampsia/eclampsia, fetal loss, prematurity, intrauterine growth restriction	SLE with APS versus PAPS in pregnancy

*Abbreviations*: *SLE* systemic lupus erythematosus, *PAPS* primary antiphospholipid syndrome, *APS* antiphospholipid syndrome

**Table 2:** Outcomes which were reported in participants with SLE versus SAPS

Studies	Outcomes reported	Types of participants
Cavallasca 2008 [16]	Stillbirth, prematurity, low for gestational age	SLE versus SAPS in pregnancy
Huong 2006 [12]	Fetal death, pre-eclampsia, arterial occlusion, arterial and venous thrombosis, premature	SLE versus SAPS in pregnancy
Ideguchi 2013 [17]	Stillbirth	SLE versus SAPS in pregnancy
Luo 2015 [18]	Preterm, fetal loss	SLE versus SAPS in pregnancy
Mecacci 2009 [19]	Preterm, low birth weight	SLE versus SAPS in pregnancy
Paramo 2002 [11]	Fetal loss	SLE versus SAPS in pregnancy
Muñoz Rodriguez 2000 [13]	Miscarriage, arterial and venous thrombosis, thrombocytopenia	SLE versus SAPS in pregnancy
Tarr 2007 [14]	Fetal loss, and thrombosis	SLE versus SAPS in pregnancy
Ko 2011 [20]	Miscarriage, stillbirth, preterm	SLE versus SAPS in pregnancy

*Abbreviations*: *SLE* systemic lupus erythematosus, *SAPS* secondary antiphospholipid syndrome

### Data extraction and quality assessment

After a careful assessment of eligibility of the respective studies, the following information was extracted/collected by two independent reviewers (PKB, and MZSS):

- The types of study reported;
- The methodological quality of the studies;
- The authors’ names and the publication year;
- The patients’ enrollment periods;
- The types of participants;
- Data relevant to the total number of pregnant women with SLE, PAPS and SAPS respectively;
- The total number of events for specific outcomes.

These data were carefully cross-checked to ensure that no data was missing. Any disagreement which followed during this data collecting was resolved by the third author (FH).

Since all the eligible studies were observational studies, quality assessment was carried out by the Newcastle Ottawa Scale (NOS) [9] using a ‘star system’ method whereby stars were given based on certain assessment criteria. A maximum total number of nine stars were possible. Higher scores indicated better qualities of the studies.

### Statistical analysis

Analytical software: RevMan version 5.3.

Statistical parameters: Risk ratios (RR) with 95% confidence intervals (CIs).

Interpretations: Heterogeneity is a major concern in meta-analyses [10]. To ensure consistency of the results, heterogeneity was assessed by the Q-statistic test whereby a *P* value less or equal to 0.05 would imply a statistically significant result. Heterogeneity was also assessed by the I^2^ statistic test with a value less than 50% representing a low level of heterogeneity and a fixed effects model was used, whereas a value above 50% indicated a higher level of heterogeneity whereby a random effects model was used.

Sensitivity analysis was carried out by an ‘exclusion method’ whereby one study was excluded each time and the results which were obtained were observed for any significant deviation.

Publication bias which was another feature often encountered in a meta-analysis, was visually interpreted using funnel plots which were generated through the RevMan software.

### Ethical approval

This is a meta-analysis and ethical or board review approval was not required.

## Results

### Searched outcomes

Following this search process, a total number of 1812 articles were obtained:

EMBASE database: 608;

MEDLINE database: 648;

Google Scholar: 527;

Official websites of specific journals which are related to rheumatology and obstetrics: 29.

Following an assessment of the titles and abstracts, which was an integral part of the eligibility criteria, 1747 articles were eliminated for irrelevancy.

Sixty-five (65) full-text articles were assessed for eligibility. However, further elimination was carried out based on the following conditions:

- Review articles (3)
- Case studies (6)
- Not related to pregnancy (7)
- Replicated/duplicated studies (39)

**Fig. 1:**
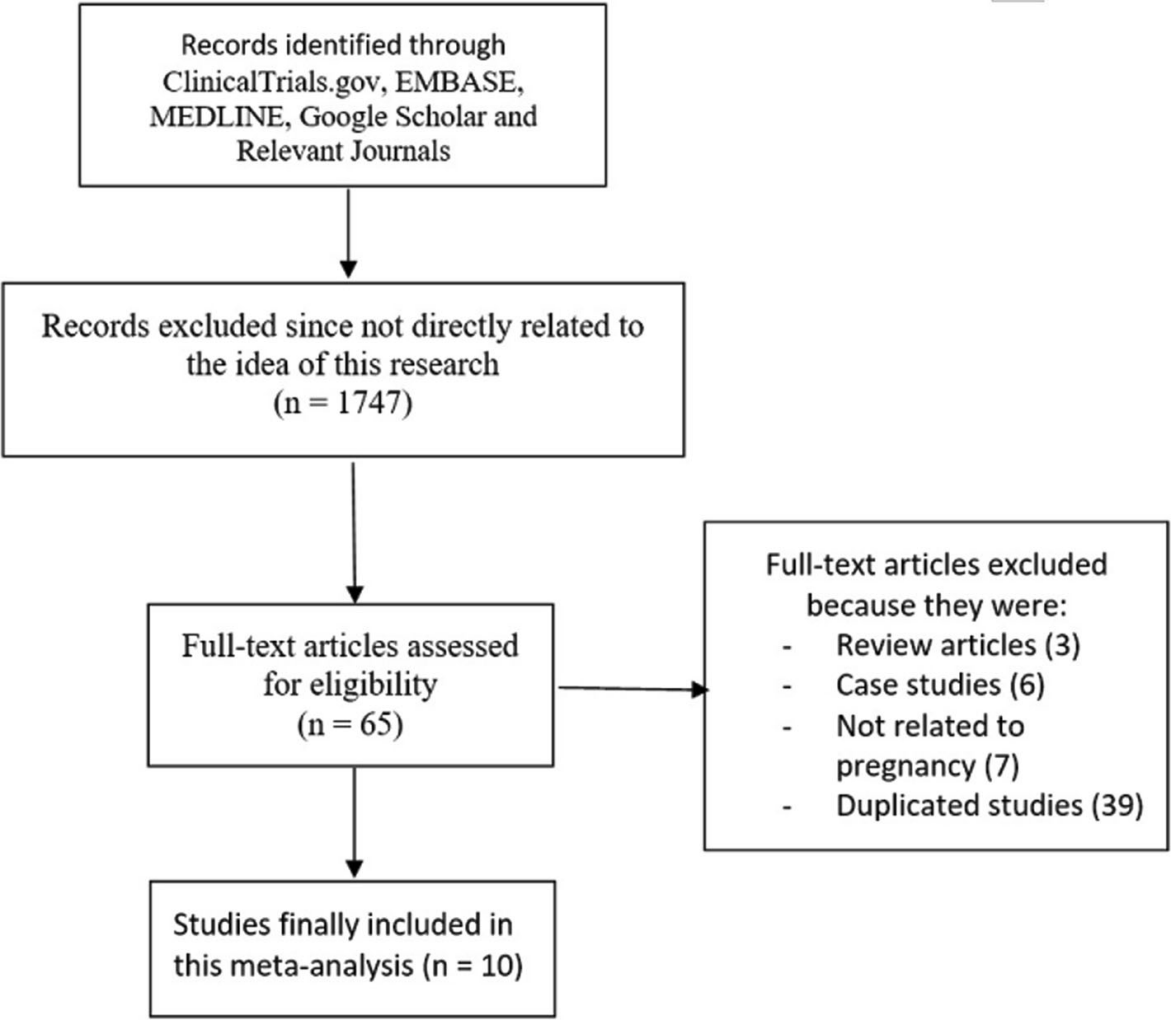
Flow diagram showing the study selection

Finally, only 10 studies [11–20] were selected for this meta-analysis as shown in Fig. 1.

The quality of the studies, which was assessed by the NOS has been shown in Table 3.

**Table 3:** Bias risk assessment with reference to the Newcastle Ottawa Scale (NOS)

Studies	Stars allotted	Total no of stars (n)	Comment on the quality of study
Paramo 2002 [11]	*******	7/9	Good
Huong 2006 [12]	********	8/9	Good
Muñoz Rodriguez 2000 [13]	******	6/9	Satisfactory
Tarr 2007 [14]	*******	7/9	Good
Cervera 2013 [15]	*******	7/9	Good
Cavallasca 2008[16]	*******	7/9	Good
Ideguchi 2013 [17]	*******	7/9	Good
Luo 2015 [18]	********	8/9	Good
Mecacci 2009[19]	******	6/9	Satisfactory
Ko 2011 [20]	*******	7/9	Good

### Main features of the eligible studies

A total number of 941 participants were included in this analysis:

- 556 pregnant women with SLE;
- 200 pregnant women with PAPS;
- 185 pregnant women with SAPS.

Type of studies: observational studies.

Patients’ enrollment period: 1986–2014 as shown in Table 4.

**Table 4:** General features of the studies

Studies	Type of study	Patients’ enrollment	No of patients with SLE (n)	No of patients with PAPS (n)	No of patients with SAPS (n)
Paramo 2002 [11]	OS	1998–2000	15	7	8
Huong 2006 [12]	OS	–	44	32	24
Muñoz Rodriguez 2000 [13]	OS	–	107	70	43
Tarr 2007 [14]	OS	–	26	26	26
Cervera 2013 [15]	OS	1990–1999	14	65	–
Cavallasca 2008 [16]	OS	1986–2004	30	–	13
Ideguchi 2013 [17]	OS	2000–2009	39	–	2
Luo 2015 [18]	OS	1990–2014	93	–	14
Mecacci 2009 [19]	OS	1998–2006	54	–	8
Ko 2011 [20]	OS	1998–2010	134	–	47
Total no of patients (n)			556	200	185

*Abbreviations*: *SLE* systemic lupus erythematosus, *PAPS* primary antiphospholipid syndrome, *SAPS* secondary antiphospholipid syndrome, *OS* observational studies

### Comparing adverse outcomes in APS versus SLE

**Fig. 2:**
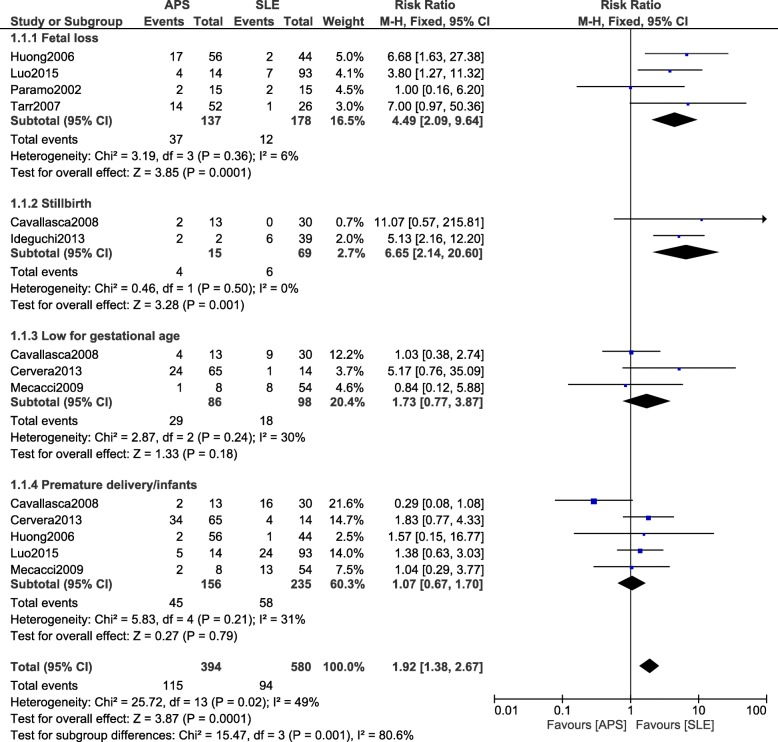
Adverse outcomes observed between APS and SLE during pregnancy

**Fig. 3:**
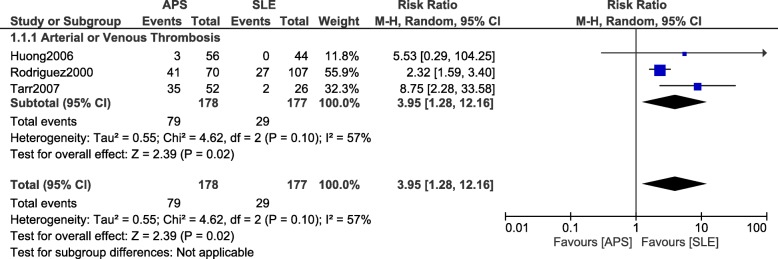
Arterial/Venous thrombosis observed between SLE and APS during pregnancy

First of all, SLE was compared with APS (PAPS and SAPS). Results of this analysis showed APS to be associated with a significantly higher risk of fetal loss (RR: 4.49, 95% CI: 2.09–9.64; *P* = 0.0001) as shown in Fig. 2. In addition, stillbirth was also significantly increased with APS (RR: 6.65, 95% CI: 2.14–20.60; *P* = 0.001). Similarly, arterial/venous thrombosis was also significantly higher in the APS group (RR: 3.95, 95% CI: 1.28–12.16; *P* = 0.02) as shown in Fig. 3. However, infants who were low for gestational age, and preterm delivery were not significantly different (RR: 1.73, 95% CI: 0.77–3.87; *P* = 0.18), (RR: 1.07, 95% CI: 0.67–1.70; *P* = 0.79) and (RR: 0.53, 95% CI: 0.10–2.76; *P* = 0.45) respectively as shown in Fig. 2.

### Comparing adverse outcomes in PAPS versus SLE

**Fig. 4:**
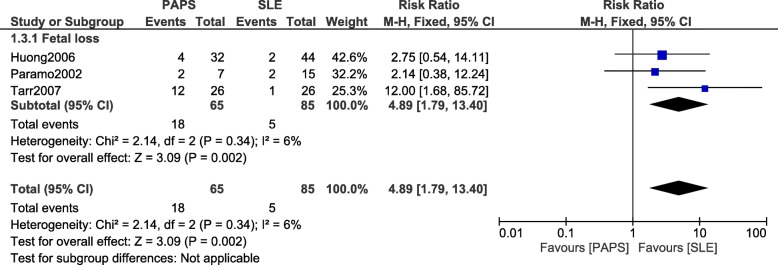
Fetal loss observed between PAPS and SLE

**Fig. 5:**
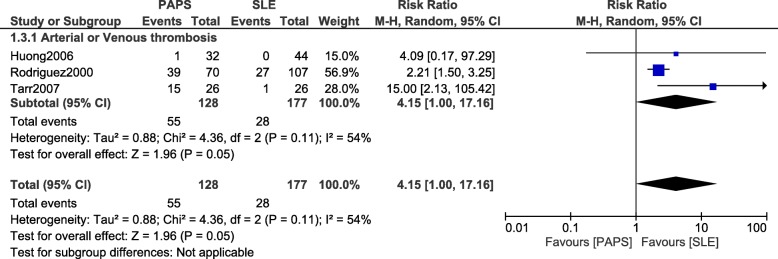
Arterial/Venous thrombosis observed between PAPS and SLE during pregnancy

When patients with PAPS were compared with patients who suffered from SLE alone, fetal loss was still significantly higher with the former (RR: 4.89, 95% CI: 1.79–13.40; *P* = 0.002) as shown in Fig. 4. Arterial/venous thrombosis was also significantly higher in patients with PAPS (RR: 4.15, 95% CI: 1.00–17.16; *P* = 0.05) (Fig. 5).

### Comparing adverse outcomes in SAPS versus SLE (without anti-phospholipid antibodies)

**Fig. 6:**
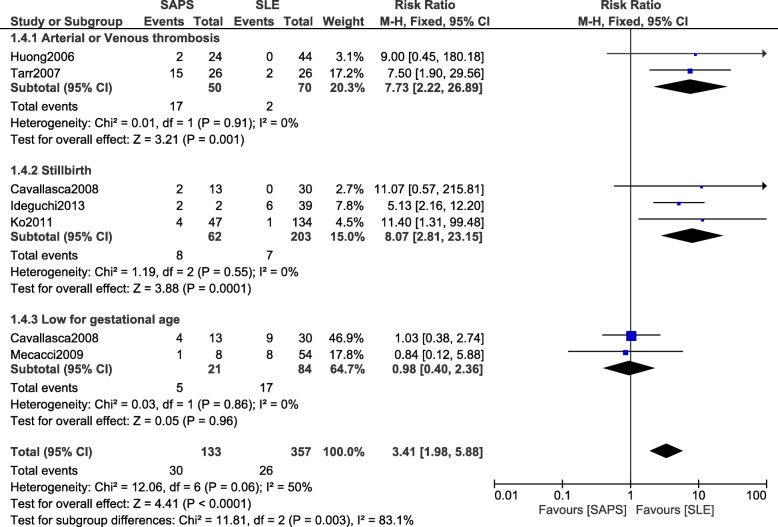
Adverse outcomes observed between SAPS and SLE during pregnancy (part 1)

When SAPS were compared with SLE, the current results showed arterial/venous thrombosis, and stillbirth to still be significantly higher with SAPS (RR: 7.73, 95% CI: 2.22–26.89; *P* = 0.001), and (RR: 8.07, 95% CI: 2.81–23.15; *P* = 0.0001) respectively (Fig. 6). However, infants who were low for gestational age were not significantly different between these two groups (RR: 0.98, 95% CI: 0.40–2.36; *P* = 0.96) (Fig. 6).

**Fig. 7:**
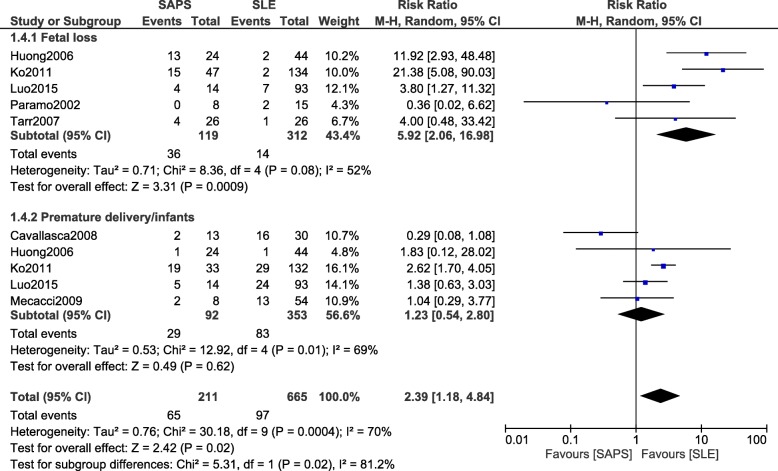
Adverse outcomes observed between SAPS and SLE during pregnancy (part 2)

Fetal loss significantly favored SLE and was therefore significantly higher in patients with SAPS (RR: 5.92, 95% CI: 2.06–16.98; *P* = 0.0009) (Fig. 7). However, premature delivery was not significantly different (RR: 1.23, 95% CI: 0.54–2.80; *P* = 0.62) (Fig. 7).

### Comparing adverse outcomes in PAPS versus SAPS

**Fig. 8:**
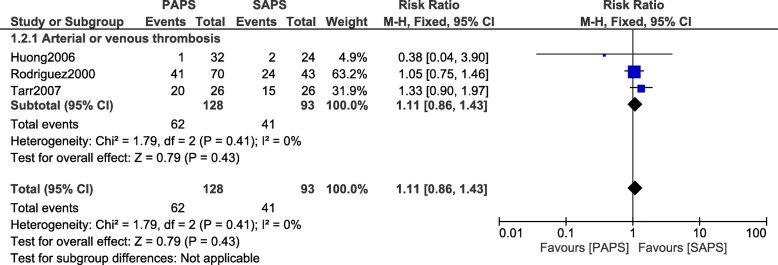
Arterial/Venous thrombosis observed between PAPS and SAPS during pregnancy

**Fig. 9:**
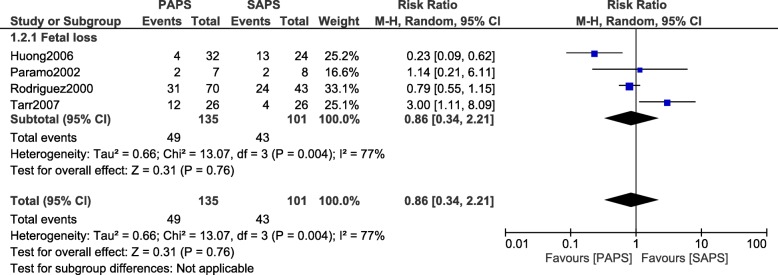
Fetal loss observed between PAPS and SAPS during pregnancy

When patients with PAPS were compared with patients who suffered from SAPS, no significant difference was observed in arterial/venous thrombosis (RR: 1.11, 95% CI: 0.86–1.43; *P* = 0.43) as shown in Fig. 8. In addition, fetal loss was also not significantly different between PAPS and SAPS (RR: 0.86, 95% CI: 0.34–2.21; *P* = 0.76) as shown in Fig. 9.

The overall results of this meta-analysis have been summarized in Table 5.

**Table 5:** Analysis of the main outcomes

Outcomes assessed	No of studies involved (n)	RR with 95% CIs	*P* value	I^2^ (%)
SLE versus APS				
Arterial and venous thrombosis	3	3.95 [1.28–12.16]	0.02	57
Fetal loss	4	4.49 [2.09–9.64]	0.0001	6
Stillbirth	2	6.65 [2.14–20.60]	0.001	0
Low for gestational age	3	1.73 [0.77–3.87]	0.18	30
Premature delivery	5	1.07 [0.67–1.70]	0.79	31
SLE versus PAPS				
Arterial and venous thrombosis	3	4.15 [1.00–17.16]	0.05	54
Fetal loss	3	4.89 [1.79–13.40]	0.002	6
SLE versus SAPS				
Arterial and venous thrombosis	2	7.73 [2.22–26.89]	0.001	0
Fetal loss	5	5.92 [2.06–16.98]	0.0009	52
Stillbirth	3	8.07 [2.81–23.15]	0.0001	0
Low for gestational age	2	0.98 [0.40–2.36]	0.96	0
Premature delivery	5	1.23 [0.54–2.80]	0.62	69
PAPS versus SAPS				
Fetal loss	4	0.86 [0.34–2.21]	0.76	77
Arterial and venous thrombosis	3	1.11 [0.86–1.43]	0.43	0

*Abbreviations*: *SLE* systemic lupus erythematosus, *APS* antiphospholipid syndrome, *PAPS* primary antiphospholipid syndrome, *SAPS* secondary antiphospholipid syndrome, *RR* risk ratios, *CIs* confidence intervals

### Sensitivity analysis and publication bias

**Fig. 10:**
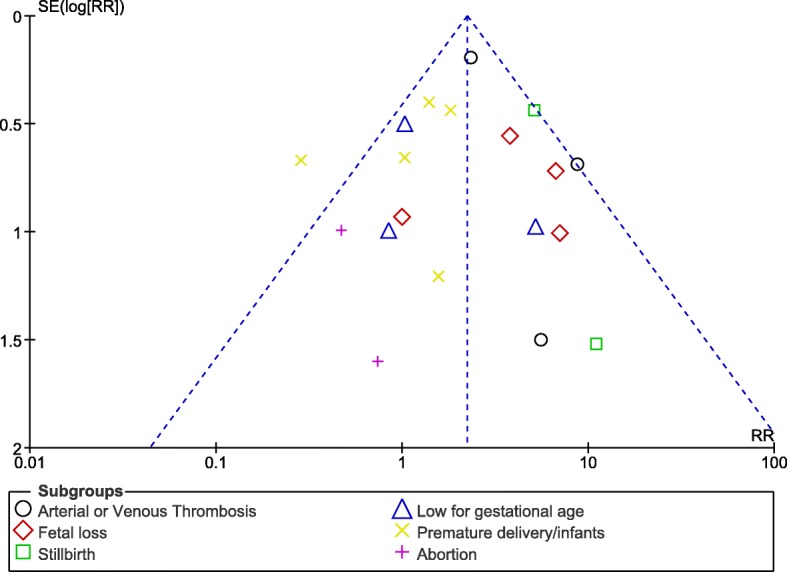
Funnel plot showing publication bias (**a**)

**Fig. 11:**
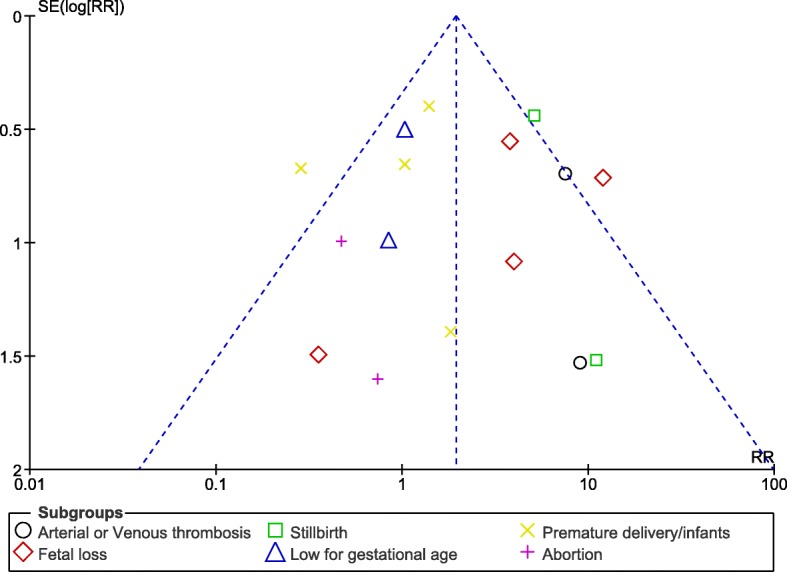
Funnel plot showing publication bias (**b**)

Sensitivity analysis which was carried out in all the sub-groups showed consistent results across the studies. Funnel plots which were graphically generated from the RevMan software, showed very little evidence of publication bias across all the studies that assessed clinical outcomes related especially to fetal loss, arterial/venous thrombosis and stillbirth as shown in Figs. 10 and 11.

## Discussion

This analysis was carried out to clarify which among SLE, PAPS and SAPS was associated with serious arterial/venous thrombosis, fetal loss and stillbirth in pregnant women. Currently, the results showed APS (PAPS and SAPS) to be associated with significantly higher maternal arterial/venous thrombosis, fetal loss and stillbirth compared to SLE. However, when PAPS and SAPS were compared with each other, no significant difference in outcomes was observed.

Apparently, SLE patients who were included in this analysis involved both active and inactive SLE during pregnancy and those patients with APS was previously diagnosed based on respective criteria [21] during their non-pregnant state. In some cases, younger women with SLE or APS do not seek medical attention in the beginning course of their disease, due to which their diagnosis remains unknown until their health conditions become unbearable. Therefore, becoming pregnant when the diagnosis of their autoimmune disorders remains unknown further contributes to high risk consequences. Despite improvement in diagnostic techniques and therapeutic options, pregnancy complications still occur in women with such autoimmune disorders and this should represent a major concern for women with similar autoimmune diseases who wish to conceive or women with similar conditions who are already pregnant [22].

Factors which might have been responsible for such outcomes in women with APS were expected to be related to APS, the disease itself. Literature reviews and other investigations showed arterial/venous thrombosis occurring due to defects in coagulation pathways to be a common feature in patients with APS [23, 24]. Umbilical artery and veins occlusion might have contributed to intrauterine fetal death. Anticardiolipin and antiphospholipid antibodies which were present in majority of patients with APS when compared to SLE, were also believed to have had a major impact on the outcomes. Recent research further showed the impact of antiphospholipid antibodies on the pathogenesis of maternal thromboembolic complications, as well as their involvement in placental insufficiency [25].

Medication use, such as low molecular weight heparin (LMWH) and low dose aspirin are considered as a beneficial therapy in pregnant women with APS [26]. In addition, use of aspirin prior to conception was associated with favorable fetal outcomes [27].

To further support these current results, Ko et al. showed that pregnancies associated with antiphospholipid antibodies were at a higher risk of adverse outcomes among the 183 pregnancies which were investigated [20]. However, it should be noted that several patients with SLE and co-existing SAPS could show an increased level of these antibodies.

When PAPS was compared with SAPS co-existing with SLE, no significant difference was observed in fetal loss and arterial/venous thrombosis. However, another study comparing thrombosis in PAPS versus APS which was associated with SLE showed the number of pregnancy loss and thrombotic events to be higher in the latter further raising controversial issues [28].

The management of pregnancy in women with SLE has recently improved drastically with the help of safer drug recommendations and assisted reproduction techniques [29, 30]. Hydroxychloroquine is also a new medication which is expected to be safe in pregnancy [31].

However, the management of pregnancy failure in patients with APS despite recommended therapies, represents a major challenge in clinics. Management of these cases is still under study. In addition to LMWH and low dose aspirin, hydroxychloroquine, has newly shown to be beneficial also to patients with antiphospholipid antibodies (PAPS and SLE with co-existing APS or SLE with antiphospholipid antibodies) [32–34]. Moreover, the global APS Score (GAPSS) might further help to predict thrombosis in those pregnant women with higher risk [35]. However, in-vitro fertilization might also be other options in patients with APS [36].

This study represents an interestingly important idea in clinical medicine. A few review articles based on this aspect were previously published, but they required the support of a meta-analysis with an evidence-based strategy just like two recently published SLE-based meta-analyses [37, 38]. Data interpretation was vital to support all these reviews of the literature. An association of APS with recurrent fetal loss and arterial/venous thrombosis was stated theoretically, however, this study has compiled and analyzed data to show evidence of this important piece of information. In addition, compared to previous works, this analysis involved a larger number of participants from different regions, showing a result which would be beyond a particular ethnic group or region, and which would therefore be relevant globally. SLE was compared with APS; PAPS and SAPS more appropriately, and PAPS was also compared to SAPS. These comparisons which have seldom been carried out systematically, and in one particular study, represent a completely new idea in clinical medicine. Even if a comparison of SLE versus APS, and PAPS versus SAPS were much more important, the comparison between SLE and SAPS was also shown.

### Limitations

The inclusion of observational data might have affected the results. However, several high-quality studies were obtained after an initial assessment of the methodological qualities, in addition to a low to moderate level of heterogeneity observed among the subgroups. Another limitation would be the restricted number of patients which might have affected the results. But it should be noted that, only a few research was carried out on this issue and therefore, only limited data were available to be used in this analysis. Another limitation was the fact that no data concerning medication use during the pregnancy stage was reported, and the influence of these medications on corresponding outcomes could not be assessed. The studies published by Tarr et al. and Munoz Rodriguez et al. showed a high number of patients with thrombosis. However, data about anti-coagulation treatments in these patients could not be extracted. A few studies showed the use of heparin and aspirin among the pregnant women. Use of these medications might also have affected the results of this analysis. In addition, a few cohorts such as the PROMISSE cohort, the French APS cohort could not be included because they involved indirect data that could not be used in this analysis. In addition, the study by Tarr et al. also included a minor number of patients who were not pregnant. This could be a limitation, however, the results were not affected due to a very small sample size of non-pregnant participants in that particular study. Also, it was possible that PAPS was a forerunner of SLE, and it remained un-noticed, and was considered as SAPS. This could have had an influence on the results too.

## Conclusions

PAPS and SAPS were associated with significantly higher arterial/venous thrombosis, fetal loss and stillbirth in comparison to SLE. However, no significant difference was observed when PAPS was compared to SAPS. This hypothesis should further be confirmed in future studies.

## Abbreviations

APS: Antiphospholipid syndrome
PAPS: Primary antiphospholipid syndrome
SAPS: Secondary antiphospholipid syndrome
SLE: Systemic lupus erythematosus
